# Lac Caninum

**Published:** 1883-07

**Authors:** E. W. Berridge

**Affiliations:** London


### CLINICAL BUREAU.

### LAC CANINUM.

E. W. Berridge, M. D.

Dec. 28th.—Miss C. G. wrote as follows: "I am suffering from very unpleasant nervous symptoms. I am not in low spirits, but I feel weak, and my nerves are so thoroughly out of order that *I cannot bear one finger to touch the other*, and often feel as though I should lose the use of my limbs. Another unpleasant symptom that I have is a sensation as if my throat were closing and I should choke. At first I fancied that this sensation was only a part of my nervous symptoms, and tried to dismiss it from my mind by being constantly employed; but now that it has steadily continued for two months, and is somewhat worse than at first, I think it will be wise to try some remedy. The sensation is between the throat and the nose. I feel as if something in the throat was either enlarged or relaxed, and I have a desire to keep my mouth open lest I should choke. Talking is very difficult, and there is a disposition to talk through the nose. When I try to swallow, sometimes I cannot, because there seems to be a kind of muscular contraction in the throat. My sleep is restless, and I frequently wake with a sick headache, *which seems to commence at the nape.* I also wake with severe pain in the lower part of the back, and am often five minutes before I can straighten myself; but this pain leaves me when I have been about my work a short time, and does not return until the next morning. My nerves have been very much overwrought, and I am afraid of being unable to perform my duties."

The italicized symptoms pointed to *Lac caninum*, and knowing that the patient was extremely sensitive to medicines, I sent her only one dose of CM (Fincke).

On January 9th she wrote as follows: "I am glad to report myself *wonderfully better*. At first the medicine made me *much worse;* my throat on the evening of the day I had taken it really seemed closing, and the difficulty of breathing was great; the nervous symptoms were worse in every way, and the eyes twitched. I had also pains in the limbs and a feeling of great mental anxiety. All this passed away in a few days, and at the present moment I feel fairly well."

I am now collecting for future publication all the pathogenetic and clinical symptoms of the *Lac* series; also all the nosodes, with the exception of *Lyssinum* and *Psorinum*, which have been already arranged by Hering and Allen. I shall be glad if those physicians who have anything to report will do so either in the pages of THE HOMŒOPATHIC PHYSICIAN, or to myself. I shall also welcome references to or quotations from any of our journals which may have escaped my notice.

